# Blisters in the Treatment of Typhoid Fever

**Published:** 1867-09

**Authors:** M. Louis


### Blisters in the Treatment of Typhoid Fever.

BY M. LOUIS.

Blisters ought to be banished from the treatment of the typhoid affection. If they exercised any influence upon the duration of the disease in the patients who have recovered, it was by prolonging it a little.

I have not only rejected vesication from the treatment of pneumonitis; I have also ceased to employ it in pleurisy and pericarditis. How can we believe that the effect of a blister is to check the inflammation, when this blister is one inflammation, superadded to another? in thoracic inflammations their usefulness is neither strictly demonstrable *nor even probable.*

One thing is most assuredly beyond question, and we should never be weary of repeating it, *that the therapeutic value of blisters is unknown;* that it must be studied by the aid of numerous and carefully noted facts, *just as if nothing at all were known about it.*

